# Cystatin C and Iris: Advances in the Evaluation of Kidney Function in Critically Ill Dog

**DOI:** 10.3389/fvets.2021.721845

**Published:** 2021-11-08

**Authors:** Fabiola de Oliveira Paes-Leme, Eliana M. Souza, Paulo Ricardo Oliveira Paes, Maderleine Geisa Gomes, Felipe Santos Muniz, Marco Túlio Gomes Campos, Renata Barbosa Peixoto, Patricia Donado Vaz de Melo, Marcio H. L. Arndt, Adriane Costa Val

**Affiliations:** ^1^Department of Clinic and Surgery, Federal University of Minas Gerais, Belo Horizonte, Brazil; ^2^Labtest Diagnóstica S.A., Lagoa Santa, Brazil; ^3^Enzytec, Biotecnologia Ltda, Belo Horizonte, Brazil

**Keywords:** dogs, cystatin C, creatinine, failure, insufficiency, illness, kidney

## Abstract

Critically ill hospitalized dogs are subject to certain complications, being acute kidney injury (AKI) a common one. Early diagnosis is crucial, and Cystatin C (CysC) is a reliable and early biomarker. The International Society of Renal Interest (IRIS) states that AKI severity can be assessed by mild changes in creatinine serum levels or reduction of urine output that cannot be considered biomarkers of renal injury but failure or insufficiency. Twenty-eight dogs admitted to the Intensive Care Unit under risk factors for the development of AKI were evaluated. Blood samples were collected for determination of sCr and CysC at admission and after 24, 48, and 72 h. Urine output was measured by daily monitoring, measured by collection in a closed system. The results showed the incidence of AKI was 67.9% based on the IRIS criteria and 78.6% based on cystatin C in critically ill patients' dogs. The measurement of serum cystatin C immediately on admission to the ICU was superior in the early identification of patients with AKI when compared to the IRIS classification and serum creatinine in critically ill dogs.

## Introduction

Acute kidney injury (AKI) is a common complication in hospitalized patients, especially in intensive care units (ICU) (1, 2). Described as the best indicator of renal function assessment, the glomerular filtration rate (GFR) has been little used in clinical practice due to its complexity (3–5) therefore, serum concentrations of urea, creatinine (sCr), and, more recently, symmetrical dimethylarginine (SDMA) are assessed more routinely.

The lack of consensual definition and uniformity for the classification system in affected dogs is challenging to diagnose AKI (6–12). In dogs, the severity of AKI is based on mild changes in serum creatinine (increases of 0.3 mg/dl in 48 h) or a reduction in the urine output (<1.0 ml/kg/h), which are no injury markers but failure or insufficiency indicators (11). Therefore, they do not allow early diagnosis, especially in assessing critical patients (13–16).

Cystatin C is a low-molecular-weight protein produced at a constant rate in all nucleated cells and excreted exclusively by the kidneys. It was identified mainly in serum, cerebrospinal fluid, kidneys, and central nervous system in dogs. This wide distribution reinforces its role in inhibiting lysosomal proteases from diseased or ruptured cells, protecting the connective tissue (17). Serum cystatin C (CysC) correlates with the glomerular filtration rate and shows an evident precocity compared to creatinine (4, 13). However, it is necessary to test it in different clinical scenarios, such as in dogs under intensive therapy and under risk factors for the development of AKI due to the critical hemodynamic state, concomitant infectious diseases, complicated surgical procedures, sepsis, or even iatrogenic causes (1, 2, 16, 18, 19). In these patients, the serum creatinine dosage has many limitations (9, 19, 20).

As there is great concern about early diagnosis of AKI so that the best therapeutic approach can be taken, the objective of this article was to perform the classification and longitudinal monitoring of renal function, as proposed by IRIS (11), and to compare this criterion with the use of CysC for the early diagnosis of renal dysfunction in dogs admitted to the intensive care unit (ICU).

## Materials and Methods

Twenty-eight dogs of different sexes, ages, and breeds admitted to the ICU of a Veterinary Hospital, who had sCr values above 1.6 mg/dl were evaluated, under risk factors for AKI development. Clinical and ultrasound examinations were added to laboratory tests for a better diagnosis of AKI.

Blood samples were collected by puncture in the external jugular vein at the time of admission to determine the sCr and CysC and after 24, 48, and 72 h, adding three samples from each animal. The collection times were chosen after evaluating the outcomes established in the 3 days of hospitalization, a protocol of the intensive care sector of the Veterinary Hospital.

According to the IRIS (12), daily monitoring of renal function was performed using sCr, monitored for 48 h for the classification of patients. The first sCr value obtained when entering the ICU was considered the baseline for each patient. For those with more than two measurements during hospitalization, the maximum sCr value was used to calculate the changes. In these dogs, sCr was considered the standard for characterizing renal function as normal (<1.6 mg/dl) or abnormal (>1.6 mg/dl). Cases of necrosis, tissue degeneration or severe myositis were ruled out as an elimination criterion to better correlate CySC with renal alterations.

Urine output was measured by daily monitoring, measured by collection in a closed system. Urinalysis was performed according to the protocol of the Veterinary Hospital Laboratory immediately after collection to ensure a fresh sample. For the urinalysis physical examination and chemical assay (URIT 14 VET) were performed, before centrifugation (5 min/450 g- LS3PLUS-Celm). After centrifugation, the supernatant was used to determine creatinine (picrate colorimetric assay- Labtest®) and protein concentration (pirogalol red colorimetric method- Labtest®) as well as gamma-glutamyl transferase activity (Szasz kinetic method- Labtest®). The urinary protein creatinine (UPC) and GGT creatinine (GGT: C) ratios were then calculated. The sediment was transferred (20 ul) to a glass slide covered (24x24- Kasvi®) and examined at low (10x) and medium (40x) power magnification under an optical microscope (CX31- Olympus®) to assess cellularity and figured elements. The CysC was measured by an immunoturbidimetric method (Cystatin C turbiquest Plus Labtest®), calibrated with canine Cystatin C. The reference CysC adopted (0.57 to 1.29 mg/L) was obtained from 19 healthy dogs, considered a control group after clinical and laboratory examination results.

To assess the performance and early detection of AKI by CysC, Spearman and Pearson linear correlation coefficient analysis was used. The significance level considered for the tests was 5%. To better understand the individual variation in serum concentrations, the % variation between times was calculated, hoping to demonstrate the magnitude of the increase.

## Results

The dogs in the ICU had a critical clinical condition, whose comorbidities, such as infectious diseases, complicated postoperative procedures, iatrogenic, and sepsis, were considered predisposing to the development of AKI, also previously described by several authors as predisposing to AKI (1, 2, 16, 18, 21, 22).

The reduction in urinary output was found in 39.3% (11/28) of the dogs analyzed; of these seven showed a reduction in the first 24 h and another four animals in the 48 h of evaluation. An increase in the urinary protein creatinine ratio (UPCR) was observed during the hospitalization (24 h = 1.04 ± 0.6; 48 h = 1.12 ± 1.27; 72 h = 1.20 ± 0.88). The urinary gamma-glutamyl transferase activity and the GGT: ratio also showed a similar increase from 24 to 48 h (125 ± 90 IU/L to 157 ± 18.9 UI/L), but lower activity at 72 h (61.9 ± 37.7 UI/L). A high prevalence of active urinary sediment (78.6%), mostly described as: erythrocytes, leukocytes, glomerular cells, and casts could be noticed, as also described by many authors (23–25).

When comparing the mean values of sCr and CysC there was no significant difference between the three analyzed times (Table 1). However, the analysis of individual values concerning the reference range showed differences and early variation of CysC. Creatinine only increased above the reference range after 48 h in seven of the 28 animals (25%), evidencing the difference in precocity between the two biomarkers.

**Table 1:** Percentage of detection of acute kidney injury (AKI) using the criteria proposed by the IRIS (12) and through the serum concentration of cystatin C in dogs during their stay in the intensive critical unit (ICU) of the Veterinary Hospital of the Federal University of Minas Gerais (UFMG).

	IRIS^*^	CYSTATIN C (≤1,29 mg/L)^**^
24 h	36,8	40,9
48 h	57,8	40,9
72 h	5,2	18,2
Total detection capability	67.8	78,6

* *IRIS AKI based on historical, serum creatinine >1.6 mg/dl or increase of 0.3 mg/dl of serum creatinine within 48 h and/or a reduction in urinary output (<1 ml/kg/h) ou anuria over 6 h,*

** *(26)*.

The individual data showed sCr increases of 0.3 mg/dl in 12 dogs (42.8%), indicating AKI after 48 h of evaluation. It was also found that the urinary flow was variable during the animals' stay in the ICU. Eleven dogs had decreased urine output, evidencing a good discriminatory capacity of this parameter. In this study, the animals were classified into three stages; the remaining 19, diagnosed with AKI, were distributed 57.8% (11/19) in stage 1, 31.5% (6/19) in stage 2, and 10.5% (2/19) in stage 3. Nine of the 28 animals evaluated (32.1%) did not show kidney damage.

Increased CysC occurred in the first 24 h while creatinine did not change. However, there was a reduction in urine output in seven animals, which made it possible to detect kidney damage using the IRIS criterion (11). The increase in CysC was progressive and associated with the worsening severity stage evidenced by the IRIS criterion after 48 h.

Based on the criteria proposed by IRIS (12), kidney injury was classified in 36.8% (7/19) of the animals at the first moment, while the CysC pointed out 40.9% (9/22) of the animals. After 48 h, there was mild azotemia in seven animals associated with reduced urine output, which allowed classification by the IRIS criterion in four of them, while CysC increased in nine animals. Finally, after 72 h, there was a reduction in the number of animals evaluated due to the limited collection of the sample or the death of some. Therefore, 16 animals were evaluated. At that time, it was observed that it was possible to diagnose only one animal with the use of the IRIS (12) criteria, while the CysC identified four animals with AKI (Table 1).

## Discussion

The animals in this study had as primary diagnosis complicated post-surgical procedures (28.6%), pyometra (14.2%), diabetic ketoacidosis (10.7%), hemoparasitosis (10.7%), encephalitis (10.7%), intoxication by non-steroidal anti-inflammatory drugs (7.1%), discospondylitis (3.6%), leishmaniasis (3.6%), pancreatitis (3.6%), periodontal disease (3.6%), and heart failure congestive (3.6%). Another observation was the occurrence of important clinical complications during their stay in the ICU, such as sepsis (53.6%), peritonitis (33.3%), hypotension (33.3%), and hypoxemia (7.4%). These etiological data are following the literature consulted, which emphasizes that AKI in the intensive care unit (ICU) has a multifactorial origin, associated with other comorbidities and complications (2, 16, 21, 27).

It was possible to observe the performance differences between CrS and CysC. Although the IRIS classification criterion is widely used (28–30) the comparison between longitudinal monitoring of CysC and sCr in critically ill dogs in the ICU has not been described in the veterinary literature so far. In this study, the system proposed by IRIS (11), which uses increased sCr and/or reduced urinary output, was used to classify and stage AKI and was compared to CysC performance.

Urine production is considered a sensitive measure for assessing renal hemodynamics, considered an early biomarker because its changes precede increases in sCr (31–33) and should always be monitored in patients with a high risk of kidney injury, as its decrease indicates the need for immediate intervention (6). It is noteworthy that patients affected by AKI, especially in the initial phase, may or may not have a decrease in the volume of diuresis. However, oliguria is a strong indicator of renal failure (6, 33). The IRIS classification reflected an increase in sCr in 42.8% (12/28) of dogs. This criterion also uses changes in urinary flow. There was a reduction of this parameter in 39.3% (11/28) of the animals. Therefore, urinary flow identified earlier stages of AKI than sCr and denoted an impaired renal function, which is generally elevated when urine output is reduced (6, 34). This fact is probably due to the low sensitivity of this evaluation in this clinical setting, where its values are greatly influenced by extra-renal factors (4, 35). Most of the dogs in this study were in stage one according to the IRIS proposal and were non-azotemic animals, which reinforces the importance of not relying on the serum creatinine reference values for the diagnosis of AKI in ICU animals partly since production, the volume of distribution and urine output are not in balance, due to the unstable conditions of the patients (14, 36, 37). In this scenario, it appears that the positive water balance, considered common in the evolution of critical, septic, and postoperative patients in which the body volume of water can increase by more than 10% in 72 h, determines a lower detectable increase in serum creatinine during the development phase of AKI. In addition, muscle loss and malnutrition associated with ICU patients reduce creatinine production, with a consequent overestimation of renal function, further delaying the diagnosis of AKI (35, 38, 39). At this stage, despite the failure to raise the serum creatinine concentration above the reference range, small increases are considered significant. This concept is important for the staging protocol, as they correspond to large reductions in the glomerular filtration rate, as mentioned by other authors (28, 37, 40). A mild to moderate azotemia was observed in other animals after 48 h of accompaniment, corroborating the literature, which reports increases in sCr values most commonly 48 to 72 after the initial insult (37, 40, 41). None of the monitored animals reached stages four and five of classification during observation in the ICU, reinforcing that therapies to prevent continuous kidney damage should be initiated even if sCr values are still within the reference ranges (29). However, it is essential to mention that, despite its slight biological variation, the application of the reference interval for sCr in dogs is not adequate alone and changes in its levels are delayed concerning impaired renal function, but it is a good marker of the evolution of renal dysfunction and its longitudinal evolution can be considered for the monitoring of AKI stages, as it guarantees the early detection of GFR decline and incipient kidney disease (4). This characteristic is considered by the system proposed by IRIS (11) in conjunction with the decrease in urine output. Thus, it is observed that the diagnosis of AKI is only made when the animals present sCr values above the reference range and, therefore, the subtle changes in sCr and diuresis still do not seem to be considered with due importance. Although the relevance of classification according to IRIS is frequently questioned and underused, its importance was demonstrated in this study.

In veterinary practice, few studies analyze the usefulness of CysC in diagnosing AKI, none of them addressing the intensive care service (4, 9). In contrast to sCr, CysC values were above the reference range in most animals assessed in the ICU. In this sense, it was observed that it detected 78.6% (22/28) of affected dogs while the criterion proposed by IRIS (12) pointed out only 67.9% (19/28) throughout the hospital stay. These data indicate a differentiated behavior of CysC and its tendency to a greater sensitivity to identify patients with AKI, which was also demonstrated before (37, 42) in evaluating severe cases. There was no significant difference between the mean values of CysC in the analysis between the different times (24, 48, and 72 h). Individual follow-up showed an increase in CysC values 24 h after the initial observation, suggesting a greater clinical impact when measured in patients on admission to the ICU. In this sense, 40.9% of the animals presented CysC values above the reference after 24 h of admission to the ICU, the same 40.9% in 48 h and 18.2% in 72 h. In comparison with CysC, none of the animals presented sCr values above the reference at admission to the ICU, although the criterion proposed by IRIS was able to identify 36.8% of patients with AKI in 24 h 57.8% after 48 h, and 5.2% in 72 h. It should be noted that considering the specificity described for CysC, minor changes should be valued, as they can represent significant changes in the clinical picture and influence the prognosis (17).

The minimum time required for diagnosing AKI using the IRIS criterion was 24 h, in addition to the need for continuous monitoring of sCr and urine output during this period, while the single dosage of CysC in ICU admission was sufficient. Therefore, the good performance of CysC and its correspondence with stages of renal dysfunction, as evidenced by the criteria recommended by IRIS, reinforce its potential precocity, sensitivity, and specificity in detecting kidney damage in critically ill dogs.

A positive and moderate correlation was observed between the levels of CysC and sCr during ICU stay (*r* = 0.44). This is possibly due to the low sensitivity of sCr in the evaluation of discrete degrees of loss of renal function, as it is only altered after 60 to 75% losses of renal function (4, 33). However, mild increases in CysC may indicate early renal failure not evidenced by sCr, which suggests that CysC may detect probable subclinical AKI. This study confirms that therapies aimed at renal protection in critically ill patients should be started immediately, even if the sCr values are within the reference range, as also evidenced elsewhere (29).

## Conclusion

The incidence of AKI was 67.9% based on the IRIS criteria and 78.6% based on Cys C, in critically ill dogs. The measurement of CysC immediately upon admission to the ICU shows superior in identifying patients with AKI compared to the IRIS classification and serum creatinine in critically ill dogs.

## Data Availability Statement

The original contributions presented in the study are included in the article/Supplementary Material, further inquiries can be directed to the corresponding author.

## Ethics Statement

The animal study was reviewed and approved by Comite de Etica em Uso de Amimais da UFMG (CEUA). Written informed consent was obtained from the owners for the participation of their animals in this study.

## Author Contributions

ES, AC, PP, MA, and FP-L conceived, designed, reviewed the project, and analyzed the data. ES, MG, MC, and RP executed the experiment and analyzed the samples. ES, AC, FM, and FP-L wrote the final manuscript. All authors contributed to the article and approved the submitted version.

## Conflict of Interest

PV was employed by company Labtest Diagnóstica S.A. MA was employed by company Enzytec, Biotecnologia Ltda. The remaining authors declare that the research was conducted in the absence of any commercial or financial relationships that could be construed as a potential conflict of interest.

## Publisher's Note

All claims expressed in this article are solely those of the authors and do not necessarily represent those of their affiliated organizations, or those of the publisher, the editors and the reviewers. Any product that may be evaluated in this article, or claim that may be made by its manufacturer, is not guaranteed or endorsed by the publisher.
