# Supplementary material for: Cystatin C and Iris: Advances in the Evaluation of Kidney Function in Critically Ill Dog
Source: Front Vet Sci. 2021 Nov 8;8:721845. doi: 10.3389/fvets.2021.721845 (PMC8606825; doi:10.3389/fvets.2021.721845)
Supplement: Supplementary file 1 [file Data_Sheet_1.DOCX]

| ANIMAL | Ciystatin C (mg∕ L) | | | Creatinine (mg∕dl) | | |
| --- | --- | --- | --- | --- | --- | --- |
|  | Hours post UTI admission | | | Hours post UTI admission | | |
|  | **24** | **48** | **72** | **24** | **48** | **72** |
| 1 | 1,27 | **1,32** | - | 1,08 | 1,20 | - |
| 2 | 1,23 | 1,28 | 1,04 | 1,11 | 1,44 | 0,72 |
| 3 | 0,95 | 0,99 | 0,92 | 0,79 | 0,71 | 0,51 |
| 4 | **1,51** | 0,96 | 1,03 | 0,41 | 0,96 | 0,6 |
| 5 | 1,11 | 1,29 | **1,33** | 0,54 | 0,94 | **1,82** |
| 6 | **1,42** | 1,28 | 1,24 | 1,20 | 0,75 | 0,77 |
| 7 | 1,19 | 1,09 | 1,20 | 1,18 | 0,81 | 0,90 |
| 8 | **1,42** | **1,48** | - | 1,43 | 1,52 | - |
| 9 | **1,38** | **1,71** | **1,46** | 1,09 | **2,41** | 1,60 |
| 10 | **1,60** | 1,28 | - | 0,89 | 0,98 | - |
| 11 | 1,10 | **3,61** | 0,71 | 0,56 | 0,12 | 0,25 |
| 12 | 0,84 | 0,80 | **1,33** | 0,94 | 0,51 | 0,83 |
| 13 | **1,52** | **1,69** | **1,85** | 1,40 | **2,06** | **1,98** |
| 14 | 1,19 | **1,37** | **1,42** | 0,57 | 0,58 | 0,94 |
| 15 | 1,16 | **1,30** | - | 0,29 | 1,09 | - |
| 16 | **1.40** | **1,34** | - | 1,35 | **1,70** | - |
| 17 | 1,18 | 1,28 | - | 0,55 | 0,98 | - |
| 18 | **1,33** | **1,43** | - | 1,57 | **3,38** | - |
| 19 | 1,10 | 1,24 | - | 1,05 | 0,71 | - |
| 20 | **1.39** | **1,42** | - | 1,44 | **1,93** | - |
| 21 | 0,98 | **1,35** | 1,10 | 0,55 | 0,13 | 0,45 |
| 22 | 1,11 | 1,27 | **1,32** | 0,61 | 0,25 | 0,20 |
| 23 | 1,24 | 1,22 | - | 1,38 | 0,94 | - |
| 24 | 1,22 | **1,31** | - | 1,28 | 1,40 | - |
| 25 | 1,28 | **1,40** | - | 1,38 | **1,77** | - |
| 26 | 0,98 | **1,38** | **1,45** | 0,65 | 0,45 | 0,35 |
| 27 | 0,75 | 0,77 | **1,39** | 0,89 | 1,04 | 0,46 |
| 28 | 0,95 | **1,55** | **1,35** | 0,59 | **3,60** | **1,77** |

- Non measured . * Reference values: creatinine < 1,6mg∕dl e Cystatine< 1,29 mg∕ L
